# Dapagliflozin combined with methylcobalamin in the treatment of type 2 diabetes mellitus with peripheral neuropathy: a systematic review and meta-analysis

**DOI:** 10.3389/fendo.2025.1514783

**Published:** 2025-06-19

**Authors:** Xiao-Long Deng, Ren Wu, Xuan-Xia Lin, Jia-Jian Liang, Han-Wei Huang

**Affiliations:** Department of Endocrinology and Metabolism, ZhongShan’s Chenxinhai Integrated Chinese and Western Medicine Hospital, Zhongshan, China

**Keywords:** diabetic peripheral neuropathy, type 2 diabetes mellitus, dapagliflozin, methylcobalamin, systematic review

## Abstract

**Background:**

Type 2 diabetes mellitus (T2DM) is a prevalent chronic metabolic disorder, with diabetic peripheral neuropathy (DPN) being one of its most common complications. Current treatments primarily aim at glycemic control and symptom relief, yet long-term efficacy is often insufficient. Dapagliflozin, an SGLT-2 inhibitor, and methylcobalamin, the active form of vitamin B12, have demonstrated potential in managing DPN. This systematic review assesses the efficacy and safety of their combined use.

**Methods:**

This study synthesized randomized controlled trials (RCTs) from major databases up to the September 30, 2024, focusing on dapagliflozin combined with methylcobalamin for treating DPN in T2DM patients. Statistical analysis was performed using RevMan 5.4. The risk of bias was assessed with the Cochrane Risk of Bias 2.0 (ROB 2.0) tool. Outcomes included the overall effective rate (OER), common peroneal motor nerve conduction velocity (CPMNCV), common peroneal sensory nerve conduction velocity (CPSNCV), median motor nerve conduction velocity (MMNCV), and median sensory nerve conduction velocity (MSNCV), fasting plasma glucose (FPG), 2-hour postprandial blood glucose (2hPG), glycosylated hemoglobin (HbA_1c_), and rate of adverse events (RAE).

**Results:**

Seven RCTs met inclusion criteria. The combination therapy significantly improved OER (odds ratio (OR): 5.05; 95% confidence interval (CI): 2.60–9.81), CPMNCV (MD: 3.93 m/s; 95% CI: 2.16–5.70; *P*<0.01), CPSNCV (MD: 3.36 m/s; 95% CI: 2.74–3.98; *P*<0.01), MMNCV (MD: 4.71 m/s; 95% CI: 3.90–5.52; *P*<0.01), and MSNCV (MD: 3.05 m/s; 95% CI: 2.19–3.90; *P*<0.01). Glycemic control also improved, with reductions in FPG (MD, 95% CI: -1.19 mmol/L [-1.40, -0.98]; *P*<0.01), 2hPG (MD, 95% CI: -1.36 mmol/L[-1.44, -1.27]; *P*<0.01), and HbA_1c_ levels (MD, 95% CI: -0.87% [-1.04, -0.71]; *P*<0.01). There was no significant increase in adverse events (OR: 0.37; 95% CI: 0.07–2.03; *P*=0.25).

**Conclusion:**

Dapagliflozin combined with methylcobalamin appears to be an effective and safe therapeutic strategy for managing DPN in T2DM patients, improving both nerve function and glycemic control.

## 1 Introduction

Type 2 diabetes mellitus (T2DM) is one of the most prevalent chronic metabolic diseases globally, marked by persistent hyperglycemia resulting from both insulin resistance and insufficient insulin secretion (1). The global prevalence of T2DM has risen rapidly, driven by lifestyle changes and the aging population (2). In 2019, an estimated 463 million people (9.3% of the world’s population) had T2DM. Projections indicate this will increase to 578 million (10.2%) by 2030 and 700 million (10.9%) by 2045 (2). Notably, urban areas exhibit higher rates of diabetes prevalence (10.8%) compared to rural areas (7.2%), and similarly, high-income countries have a greater burden (10.4%) compared to low-income countries (4.0%) (2). These figures highlight the increasing global burden of T2DM, especially in urban and high-income regions, emphasizing the urgent need for effective prevention and treatment strategies to combat this expanding public health issue.

Diabetic peripheral neuropathy (DPN) is one of the most common and debilitating complications of T2DM. It is a complex condition that arises from multiple pathogenic mechanisms, including hyperglycemia-induced neuronal injury, vascular dysfunction, oxidative stress, and inflammation (3). Clinically, DPN is characterized by symptoms such as numbness, tingling, burning pain, and sensory impairment, primarily affecting the extremities (4). In advanced cases, DPN may lead to ulcers, infections, and even amputations, severely impacting the quality of life (4). It is estimated that up to 50% of individuals with T2DM will develop DPN during their lifetime, underscoring the significant burden of this condition (3).

Currently, treatment strategies for DPN focus on glycemic control, symptomatic pain relief, and neurotrophic support (5). While tight glycemic control can slow the progression of neuropathy, it does not reverse the established nerve damage (5). Analgesics such as antidepressants, anticonvulsants, and opioids are commonly prescribed to alleviate pain, but these treatments are often associated with adverse effects and limited long-term efficacy (5). Methylcobalamin, a biologically active form of vitamin B12, has been shown to support nerve repair and regeneration; however, its therapeutic effectiveness as monotherapy is often insufficient to provide comprehensive relief from neuropathic symptoms (6, 7).

Dapagliflozin, a sodium-glucose co-transporter 2 (SGLT-2) inhibitor, has been increasingly recognized for its role in reducing blood glucose levels by promoting glucose excretion through the urine (8). In addition to its glucose-lowering effect, dapagliflozin offers other benefits such as weight reduction, improved blood pressure control, and cardiovascular and renal protection (9). Emerging research suggests that SGLT-2 inhibitors may also possess neuroprotective properties, potentially through anti-inflammatory and metabolic regulatory pathways, making them a promising option for the treatment of DPN (8).

Current clinical guidelines from the American Diabetes Association and European Association for the Study of Diabetes do not yet recommend combining dapagliflozin and methylcobalamin as a standard treatment for DPN. However, growing evidence supports their individual therapeutic benefits (10). Dapagliflozin has demonstrated efficacy for glycemic control and cardiovascular protection in T2DM patients (8, 9), while methylcobalamin plays a well-established role in nerve repair and regeneration (11). The combination of these agents may offer a synergistic approach by simultaneously addressing both the metabolic dysfunction through dapagliflozin’s glucose-lowering effects and structural nerve damage through methylcobalamin’s neurotrophic actions. While preliminary clinical evidence appears promising, further rigorous research is required to establish the efficacy and safety of this combination therapy before it can be incorporated into formal treatment guidelines.

The objective of this study is to evaluate the efficacy and safety of combining dapagliflozin with methylcobalamin in the management of peripheral neuropathy in patients with T2DM. By comparing the clinical outcomes of the combination therapy to other modalities, we aim to determine whether the combined treatment is more effective in alleviating neuropathic symptoms, improving nerve conduction, and enhancing blood glucose levels. Furthermore, the safety and tolerability of the combination regimen were assessed, providing critical insights for the clinical management of DPN.

## 2 Methods

### 2.1 Study design

This systematic review was conducted in accordance with the Preferred Reporting Items for Systematic Reviews and Meta-Analyses guidelines (12). The study protocol was registered in the International Platform of Registered Systematic Review and Meta-analysis Protocols (INPLASY) with the registration number ID: INPLASY202530084; and DOI number is 10.37766/inplasy2025.3.0084; and website of file:///C:/Users/HW/Downloads/INPLASY%20Protocol%207589.pdf.

This study utilizes a systematic review approach to synthesize findings from multiple randomized controlled trials (RCTs) to derive a more accurate estimate of the overall treatment effect of dapagliflozin combined with methylcobalamin in treating T2DM patients with DPN. The methodology includes a comprehensive literature search, data extraction, quality assessment, and statistical analysis to integrate the results from the included RCTs.

### 2.2 Literature search strategy

A comprehensive literature search was conducted across several databases, including PubMed, Embase, Cochrane Library, and China National Knowledge Infrastructure (CNKI), covering studies from the inception of each database to the September 30, 2024. Search terms included ‘dapagliflozin,’ ‘methylcobalamin,’ ‘type 2 diabetes mellitus,’ and ‘peripheral neuropathy’. No language restrictions were applied. Additional sources were identified through reference lists of the included studies and clinical trial registries, ensuring the comprehensiveness of the search. The detailed search strategy is provided in **Supplementary Table S1**.

### 2.3 Inclusion and exclusion criteria

The systematic review included studies based on the following criteria: (1) only RCTs were selected to ensure high-quality evidence; (2) studies that involved patients diagnosed with T2DM complicated by DPN; (3) studies where the intervention consisted of dapagliflozin combined with methylcobalamin; and (4) studies that reported outcomes related to neuropathic symptom improvement, nerve conduction velocity, blood glucose levels, and adverse events. Exclusion criteria were: (1) studies that did not employ a randomized controlled design; (2) studies that did not include both dapagliflozin and methylcobalamin as part of the treatment regimen; and (3) studies with incomplete or unavailable data.

### 2.4 Data extraction

A standardized data extraction form was developed to ensure consistency across studies. The extracted data included: (1) basic study information such as title, authors, publication year, and journal; (2) patient characteristics such as age, gender, duration of diabetes, and baseline neuropathy status; (3) intervention details, including dosage and duration of dapagliflozin and methylcobalamin; (4) efficacy outcomes, including overall effective rate (OER), motor nerve conduction velocity (MNCV, m/s), sensory nerve conduction velocity (SNCV,m/s), and blood glucose levels. Specific measurements for nerve conduction velocity included common peroneal motor nerve conduction velocity (CPMNCV, m/s), common peroneal sensory nerve conduction velocity (CPSNCV, m/s), median motor nerve conduction velocity (MMNCV, m/s), and median sensory nerve conduction velocity (MSNCV, m/s). Blood glucose measurements included fasting plasma glucose (FPG, mmol/L), 2-hour postprandial blood glucose (2hPG, mmol/L), and glycosylated hemoglobin (HbA_1c_, %). (5) Safety outcomes were assessed based on the reported rate of adverse events (RAE).

### 2.5 Risk of bias assessment

The methodological quality and risk of bias in the included RCTs were systematically evaluated using the Cochrane Risk of Bias 2.0 (ROB 2.0) tool (13). This tool provides a structured approach to evaluate potential biases across five core domains, each addressing critical aspects of trial design and conduct. First, the randomization process was assessed to determine whether the allocation sequence was generated and implemented appropriately, ensuring comparable baseline characteristics between groups. Second, deviations from intended interventions were examined to identify any systematic differences in care provided apart from the intervention under investigation. Third, missing outcome data were analyzed to evaluate the extent and potential impact of participant attrition or exclusion on the study results. Fourth, the measurement of outcomes was scrutinized to assess the validity and reliability of outcome assessment methods, including the adequacy of blinding procedures. Finally, the selection of reported results was reviewed to detect any potential bias arising from selective reporting of outcomes or analyses. For each domain, a series of signaling questions guided the evaluation, and an overall risk of bias judgment (low risk, some concerns, or high risk) was assigned based on predefined criteria. This comprehensive assessment framework not only enhances the transparency and reproducibility of our evaluation but also provides a more nuanced understanding of the methodological strengths and limitations of the included studies, thereby strengthening the validity of our findings.

### 2.6 Certainty of evidence

The certainty of evidence was evaluated using the Grading of Recommendations, Assessment, Development, and Evaluation (GRADE) framework (14). According to this methodology, evidence from the included studies was initially classified as “high quality” but could be downgraded to “moderate quality,” “low quality,” or “very low quality” based on the presence and severity of five key limitations: risk of bias, indirectness, inconsistency, imprecision, and publication bias. A summary of findings table was created to present and interpret the final results. Two independent reviewers, who also conducted the risk of bias assessment, were responsible for applying the GRADE approach to ensure consistency and rigor in the evaluation process.

### 2.7 Statistical analysis

Data from the included studies were pooled using RevMan 5.4 (The Cochrane Collaboration) for statistical analysis. For dichotomous outcomes, odds ratio (OR) was calculated, while mean difference (MD) was used for continuous outcomes. The heterogeneity among studies was assessed using the *I*² statistic and the *Q* test (15). An *I*² value greater than 50% or a significant *Q* test (*P* < 0.10) indicated substantial heterogeneity, warranting the use of a random-effects model. If heterogeneity was low, a fixed-effects model was applied.

## 3 Results

### 3.1 Literature search and screening results

**Figure 1:**
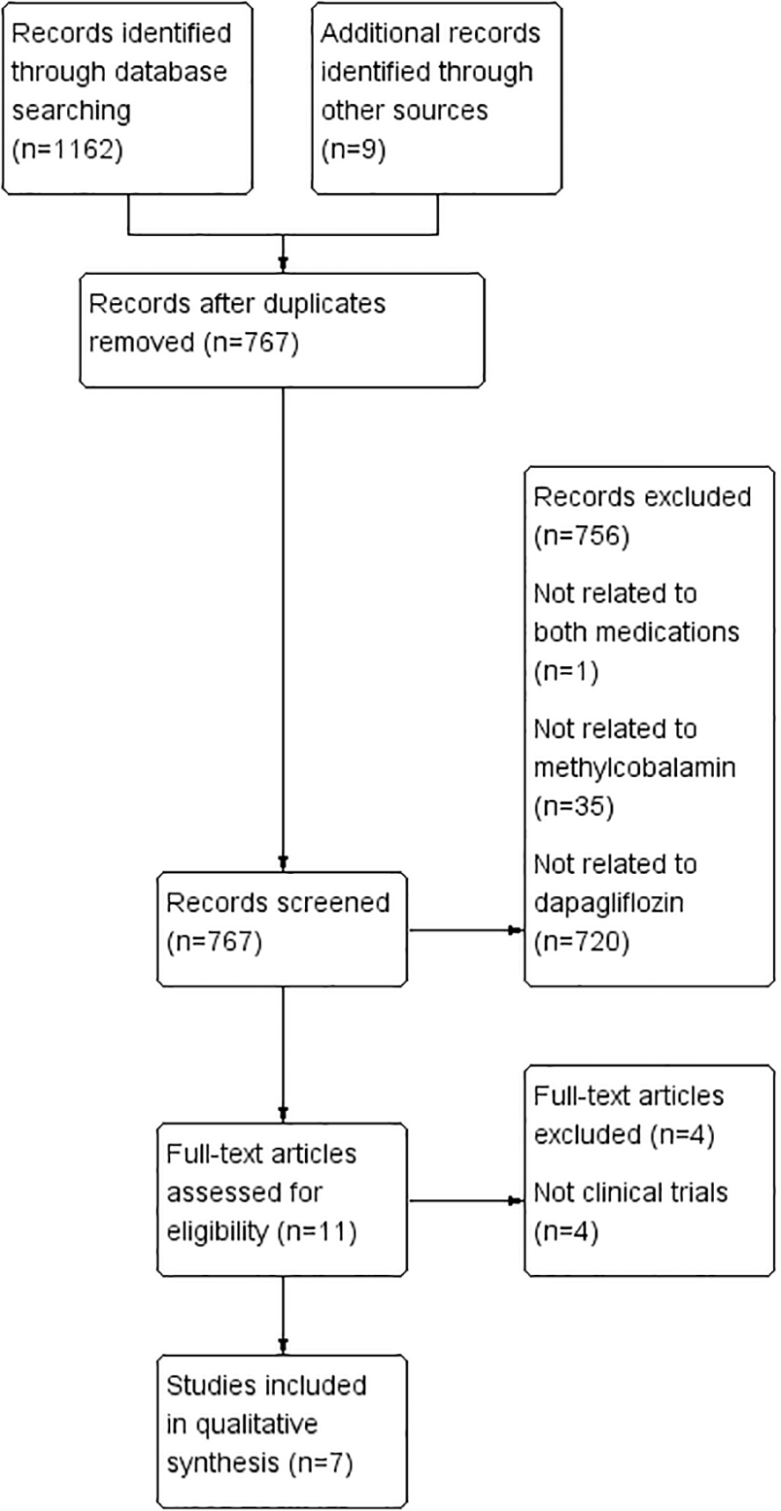
Flowchart of study selection.

A comprehensive literature search across PubMed, Embase, Cochrane Library, CNKI, and Wanfang databases identified 1,171 articles. After eliminating duplicates, 767 articles were retained for initial screening based on titles and abstracts. Of these, 11 articles were deemed potentially relevant and underwent full-text review. Following a thorough assessment, seven RCTs met the inclusion criteria and were included in the systematic review (16–22) (**Figure 1**).

### 3.2 Characteristics of included studies

The characteristics of the included studies are summarized in **Table 1** (16–22). The studies, published between 2017 and 2023, had sample sizes ranging from 50 to 100 participants, with a total of 2,450 patients. The mean age of the participants was 58 years, and there was a slight male predominance across the studies. All studies involved patients diagnosed with T2DM and DPN, and the intervention consisted of dapagliflozin combined with methylcobalamin. The treatment duration varied between 12 and 52 weeks.

**Table 1:** General characteristics of included studies.

Study	Country	No. of patients (T/C)	Age (years, T/C)	Gender (M/F)	Duration	Treatment (dosage)	Control	Outcome measures
Hu 2020 (16)	China	50/50	T:58.62±5.81; C:58.27±5.72	T: 29/21 C:28/22	1 month	Dapagliflozin (5 mg, qd), plus Mecobalamin (0.5mg, tid)	Metformin(0.5g,tid) plus Mecobalamin (0.5mg, tid)	OER↑, CPMNCV↑, CPSNCV↑, MMNCV↑, MSNCV↑, FPG↓, 2hPG↓, HbA_1c_↓
Li 2020 (17)	China	40/40	T:50.0±2.60; C:50.0±3.70	T: 20/20 C:19/21	4 weeks	Dapagliflozin (5 mg, qd), plus Mecobalamin (0.5mg, tid)	Metformin(0.5g,tid) plus Mecobalamin (0.5mg, tid)	OER↑, CPMNCV↑, CPSNCV↑, MMNCV↑, MSNCV↑
Shi 2019 (18)	China	20/20	T:61.75±5.24; C:60.85±4.39	T: 8/12 C:11/9	4 weeks	Dapagliflozin (5 mg, qd), plus Mecobalamin (0.5mg, tid)	Metformin(0.5g,tid) plus Mecobalamin (0.5mg, tid)	CPMNCV↑, CPSNCV↑, MMNCV↑, MSNCV↑
Sun 2023 (19)	China	45/43	T:57.79±8.14; C:58.94±8.25	T: 22/23 C:21/22	NR	Dapagliflozin (5 mg, qd), plus Mecobalamin (0.5mg, tid)	Metformin(0.5g,tid) plus Mecobalamin (0.5mg, tid)	OER↑, FPG↓, 2hPG↓, HbA_1c_↓
Wu 2021 (20)	China	44/44	T:64.2±3.3; C:63.9±3.0	T: 28/16 C:26/18	4 weeks	Dapagliflozin (5 mg, qd), plus Mecobalamin (0.5mg, tid)	Metformin(0.5g,tid) plus Mecobalamin (0.5mg, tid)	OER↑, CPMNCV↑, CPSNCV↑, MMNCV↑, MSNCV↑, FPG↓, 2hPG↓, HbA_1c_↓, RAE
Xiong 2018 (21)	China	25/25	T:40.89±0.38; C:40.67±0.29	T: 13/12 C:15/10	NR	Dapagliflozin (5 mg, qd), plus Mecobalamin (0.5mg, tid)	Metformin(0.5g,tid) plus Mecobalamin (0.5mg, tid)	CPMNCV↑, CPSNCV↑, MMNCV↑, MSNCV↑
Yang 2017 (22)	China	41/41	T:59.6±4.3; C:62.5±3.5	T: 20/21 C:22/19	4 weeks	Dapagliflozin (5 mg, qd), plus Mecobalamin (0.5mg, tid)	Metformin(0.5g,tid) plus Mecobalamin (0.5mg, tid)	OER↑, CPMNCV↑, CPSNCV↑, MMNCV↑, MSNCV↑, FPG↓, 2hPG↓, HbA_1c_↓

T, treatment group; C, control group; M, male; F, female; qd, once daily; tid, three times daily; NR, not report; OER, overall effective rate; CPMNCV, common peroneal motor nerve conduction velocity; CPSNCV, common peroneal sensory nerve conduction velocity; MMNCV, median motor nerve conduction velocity; MSNCV, median sensory nerve conduction velocity; FPG, fasting blood.

↓means decrease, ↑means increase.

### 3.3 Quality assessment

The results of risk of bias were evaluated by ROB 2.0 tool (**Supplementary Figure S1**). Five studies demonstrated a low risk of bias in random sequence generation. Additionally, all studies exhibited a low risk of bias regarding incomplete outcome data, selective reporting, and other biases. However, several studies did not adequately report on allocation concealment, blinding of participants and personnel, and outcome assessment, leading to an unclear risk of bias in these domains.

### 3.4 Efficacy analysis

The efficacy of dapagliflozin combined with methylcobalamin in the treatment of DPN was evaluated through a meta-analysis. The results demonstrated significant improvements in neuropathic symptoms and nerve function. The pooled effect sizes showed significant differences in OER (OR, 95% confidence interval (CI): 5.05 [2.60, 9.81]; *P*<0.01), CPMNCV (MD, 95% CI: 3.93m/s [2.16, 5.70]; *P*<0.01), CPSNCV (MD, 95% CI: 3.36 m/s [2.74, 3.98]; *P*<0.01), MMNCV (MD, 95% CI: 4.71m/s [3.90, 5.52]; *P*<0.01), and MSNCV (MD, 95% CI: 3.05m/s [2.19, 3.90]; *P*<0.01). Additionally, the combination therapy resulted in significant improvements in FPG (MD, 95% CI: -1.19 mmol/L [-1.40, -0.98]; *P*<0.01), 2hPG (MD, 95% CI: -1.36 mmol/L [-1.44, -1.27]; *P*<0.01), and HbA_1c_ levels (MD, 95% CI: -0.87% [-1.04, -0.71]; *P*<0.01), indicating a positive effect on both nerve function and glycemic control (**Supplementary Figures S2**-**S9**, **Supplementary Table S2**).

Sensitivity analyses revealed that dapagliflozin and methylcobalamin exhibited comparable overall efficacy in the treatment of diabetic peripheral neuropathy (DPN). Both treatments demonstrated superior improvements in CPMNCV (MD, 95% CI: 3.62 m/s [3.02, 4.23]; *P*<0.01), CPSNCV (MD, 95% CI: 3.70 m/s [3.30, 4.09]; *P*<0.01), MMNCV (MD, 95% CI: 5.86 m/s [4.90, 6.82]; *P*<0.01), MSNCV (MD, 95% CI: 3.67 m/s [2.99, 4.34]; *P*<0.01), and FPG (MD, 95% CI: -1.26 mmol/L [-1.49, -1.03]; *P*<0.01). These findings suggest that the combination of dapagliflozin and methylcobalamin retains distinct advantages in enhancing nerve function and glycemic control, even after sensitivity analyses (**Supplementary Figures S3**-**S7**).

### 3.5 Safety analysis

The safety profile of dapagliflozin combined with methylcobalamin was assessed by analyzing the adverse events reported in the included studies. The results indicated that there was no significant increase in the risk of adverse events associated with the combination therapy compared to the control groups. The pooled OR for adverse events was 0.37 (95% CI: 0.07 to 2.03; *P*=0.25), suggesting that the combination of dapagliflozin and methylcobalamin was well-tolerated and did not raise safety concerns (**Supplementary Table S2**).

### 3.6 Results of certainty of evidence

The certainty of evidence for each outcome, as assessed using the GRADE framework, is summarized in **Supplementary Table S3**. In this study, the certainty of evidence for all outcomes was downgraded by at least one level due to imprecision, primarily because the total number of participants for each outcome was fewer than 100. Additionally, some outcomes were further downgraded by two levels due to significant intrastudy inconsistency.

## 4 Discussion

### 4.1 Main findings

This systematic review demonstrated that dapagliflozin combined with methylcobalamin significantly improves neuropathic symptoms in patients with T2DM complicated by DPN. The pooled data revealed notable enhancements in nerve conduction velocity and substantial reductions in blood glucose level. These results suggest that the combination therapy not only alleviates blood glucose but also enhances nerve function. Furthermore, the analysis confirmed that this combination is well-tolerated, with no significant increase in adverse events compared to control treatments. The most frequently reported side effects were mild gastrointestinal symptoms, which were manageable and did not necessitate discontinuation of the therapy.

### 4.2 Comparison with previous studies

The findings of this review are consistent with prior studies on the individual effects of SGLT-2 inhibitors and vitamin B12 analogs in diabetic neuropathy (23–27). Previous meta-analyses and large-scale RCTs have documented similar improvements in neuropathic symptoms when dapagliflozin or methylcobalamin were used independently (28). However, this study is among the first to systematically evaluate the combined effects of these agents. The synergistic benefits observed in this review can likely be attributed to their complementary mechanisms of action: dapagliflozin improves glycemic control and reduces oxidative stress, while methylcobalamin supports nerve repair and regeneration.

The observed benefits of combining Dapagliflozin and Methylcobalamin can be attributed to their complementary mechanisms of action. Dapagliflozin improves glycemic control by promoting urinary glucose excretion, thereby reducing hyperglycemia-induced oxidative stress and inflammation, which are key contributors to nerve damage in DPN (8). Additionally, Dapagliflozin has been shown to improve endothelial function and microvascular circulation, further protecting against neuropathy (8, 9). Methylcobalamin, the active form of vitamin B_12_, supports nerve repair and regeneration by promoting myelin synthesis and reducing homocysteine levels, which are known to exacerbate neuropathic damage (11). Together, these agents address both the metabolic and structural components of DPN, offering a comprehensive therapeutic approach. Recent studies have further supported the role of vitamin B_12_ analogs in managing diabetic neuropathy. A systematic review by Lekhanya et al. highlighted the potential of vitamin B_12_ complex in improving neuropathic symptoms and nerve function in diabetic patients, reinforcing the importance of methylcobalamin in nerve repair and regeneration (29).

Emerging evidence underscores the multifaceted role of SGLT2 inhibitors in addressing peripheral neuropathy, particularly as a key component in the pathophysiology of diabetic foot. Preclinical and clinical studies have demonstrated that SGLT2 inhibitors, such as dapagliflozin, exert neuroprotective effects through mechanisms that include anti-inflammatory actions, reduction of oxidative stress, and improvement of endothelial function (30, 31).These agents not only enhance glycemic control but also exhibit pleiotropic benefits, such as improving microvascular circulation and reducing arterial stiffness, which are critical in mitigating the progression of diabetic neuropathy (30). Early intervention with SGLT2 inhibitors, combined with their pleiotropic properties, may significantly contribute to delaying or preventing the onset of diabetic foot, a severe and debilitating complication of diabetic peripheral neuropathy (31). These findings highlight the potential of SGLT2 inhibitors as a comprehensive therapeutic strategy for managing DPN and its associated complications, offering both symptomatic relief and long-term protective benefits.

### 4.3 Possible reasons for differences in results

Variations in study populations, treatment durations, and outcome measures may explain the differences observed between this review and previous studies (32, 33). For instance, studies with longer treatment durations demonstrated greater improvements in nerve conduction velocity, suggesting that prolonged therapy is more effective for nerve repair. Additionally, differences in the baseline severity of neuropathy among participants could influence treatment outcomes, with more pronounced benefits seen in patients with moderate to severe neuropathy (32). Methodological quality also plays a role, as higher-quality studies tend to produce more reliable results.

### 4.4 Clinical significance

The findings of this review have important implications for clinical practice. Dapagliflozin combined with methylcobalamin appears to be a safe and effective therapeutic option for managing DPN in patients with T2DM. Improvements in nerve conduction velocity and blood glucose levels suggest that this combination therapy can significantly improve patients’ quality of life by reducing neuropathic symptoms, enhancing nerve function, and decreasing blood glucose levels. Clinicians may consider integrating this combination into treatment protocols, especially for patients who have not achieved adequate relief from other therapies. The favorable safety profile also supports the broader application of this therapy, including in patients with comorbid conditions.

### 4.5 Implications for future research

While this systematic review offers valuable insights into the clinical application of dapagliflozin and methylcobalamin, further research is essential to optimize their therapeutic potential and address existing evidence gaps. Future investigations should prioritize the following key areas to refine clinical practice and strengthen the evidence base.

Future studies should focus on identifying the most effective dosing regimens and treatment durations to maximize therapeutic outcomes, including exploring dose-response relationships and individualized dosing strategies. Mechanistic research is needed to elucidate the underlying pathways through which dapagliflozin and methylcobalamin exert synergistic effects, particularly their roles in reducing oxidative stress, mitigating inflammation, and improving microvascular function (34). Long-term, large-scale clinical trials are necessary to assess the sustainability of therapeutic benefits and monitor potential delayed adverse events, with extended follow-up periods to evaluate durability of symptom relief and safety profiles. Additionally, research should expand to include diverse patient populations across age groups, ethnicities, and stages of neuropathy severity to enhance generalizability and identify subgroup-specific responses. Addressing these priorities will refine the scientific understanding of this combination therapy and optimize its clinical utility in managing DPN.

### 4.6 Study limitations

This systematic review has several limitations that should be acknowledged. First, the quality of the included studies varied, with some exhibiting unclear or high risks of bias in key domains, which may affect the reliability of the pooled results. Second, moderate to high heterogeneity was observed in the efficacy outcomes, indicating variability in treatment effects across studies. Third, the reliance on reported data introduces the potential for reporting bias, as not all studies provided comprehensive information on adverse events or secondary outcomes. Fourth, the generalizability of the findings may be limited by the characteristics of the included studies, which were largely conducted in specific geographic regions and involved middle-aged adults with moderate to severe neuropathy. Additional research is needed to confirm the applicability of these results to other populations, including older adults and those with milder forms of neuropathy. Finally, the small number of included studies may limit the statistical power of our meta-analysis. While the pooled results suggest significant improvements in nerve function and glycemic control, the limited sample size may affect the generalizability of our findings. Future research with larger sample sizes is needed to confirm these results.

## 5 Conclusion

This systematic review provides robust evidence that the combination of dapagliflozin and methylcobalamin is an effective and safe therapeutic strategy for managing DPN in patients with T2DM. The combination therapy significantly improves nerve conduction velocities, reduces blood glucose levels, and demonstrates a favorable safety profile, with adverse events comparable to control treatments. These findings underscore the potential of this combination therapy to enhance current treatment regimens for DPN, offering both symptomatic relief and functional recovery for patients.

## Data Availability

All original data and findings from this study are presented within the article. For further information, inquiries can be directed to the corresponding author.
