# Supplementary material for: Dapagliflozin combined with methylcobalamin in the treatment of type 2 diabetes mellitus with peripheral neuropathy: a systematic review and meta-analysis
Source: Front Endocrinol (Lausanne). 2025 Jun 19;16:1514783. doi: 10.3389/fendo.2025.1514783 (PMC12221928; doi:10.3389/fendo.2025.1514783)
Supplement: Supplementary file 1 [file DataSheet1.pdf]

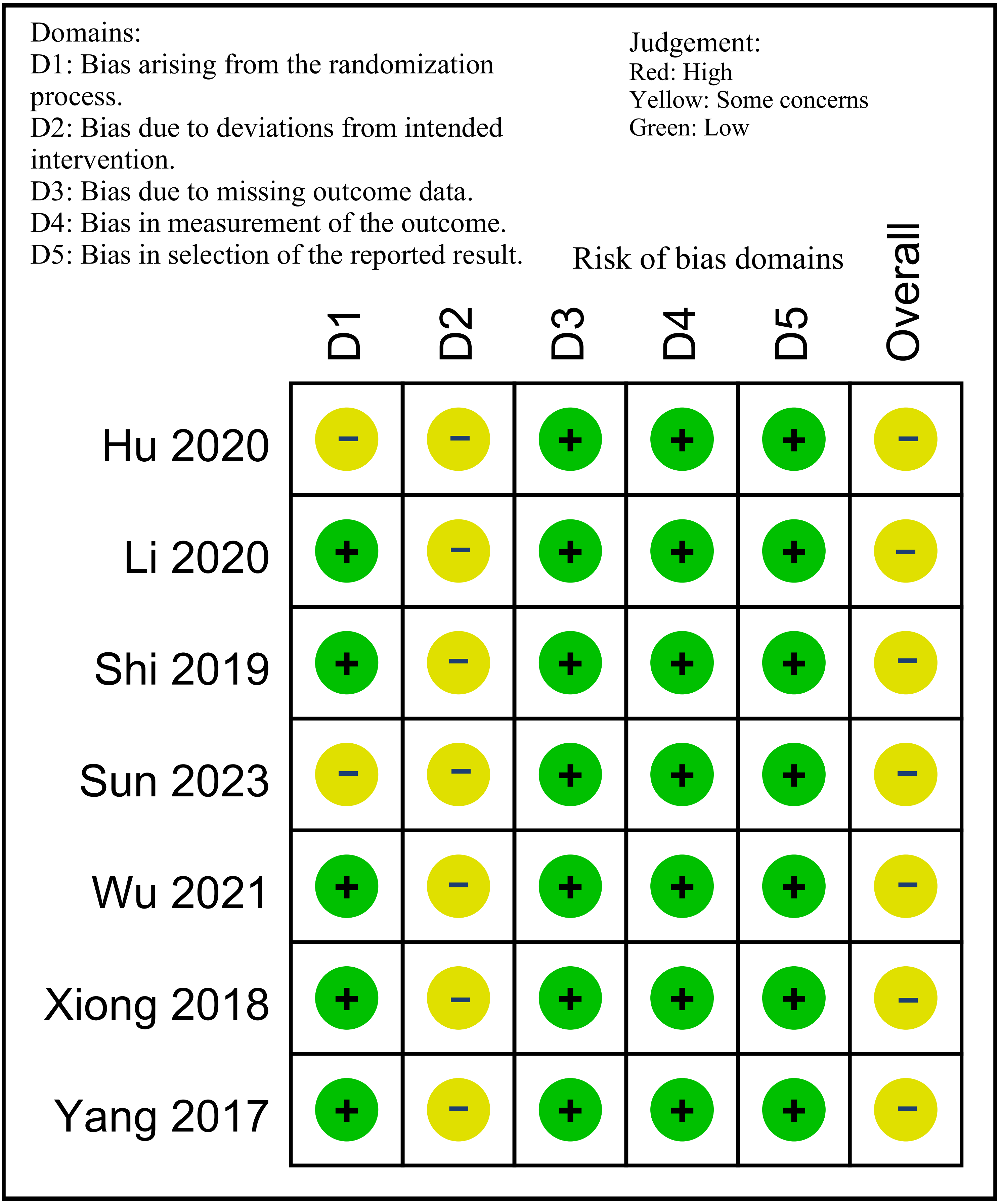

Supplementary Figure S1. Risk of bias of included studies

Study or Subgroup	Experimental Events	Experimental Total	Control Events	Control Total	Weight	Odds Ratio M-H, Fixed, 95% CI
Hu 2020	49	50	40	50	8.7%	12.25 [1.50, 99.80]
Li 2020	38	40	30	40	16.3%	6.33 [1.29, 31.11]
Sun 2023	42	45	33	43	24.4%	4.24 [1.08, 16.67]
Wu 2021	41	44	33	44	24.4%	4.56 [1.17, 17.69]
Yang 2017	38	41	33	41	26.2%	3.07 [0.75, 12.53]
**Total (95% CI)**		**220**		**218**	**100.0%**	**5.05 [2.60, 9.81]**
Total events	208		169			

Heterogeneity: $Chi^2 = 1.33$, df = 4 (P = 0.86); $I^2 = 0\%$

Test for overall effect: Z = 4.77 (P < 0.00001)

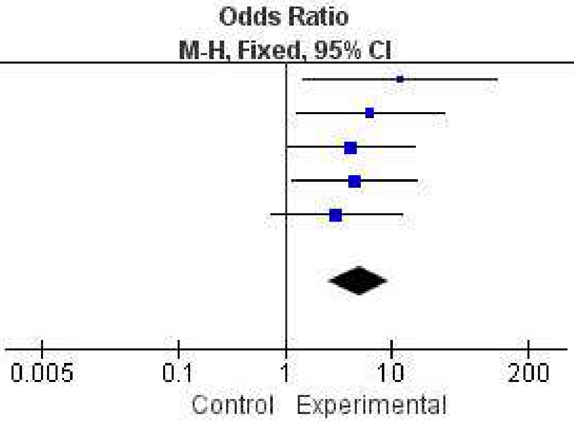

Supplementary Figure S2. Meta-analysis of OER

Study or Subgroup	Experimental Mean	Experimental SD	Experimental Total	Control Mean	Control SD	Control Total	Weight	Mean Difference IV, Random, 95% CI
**1.2.1 Common peroneal motor nerve conduction velocity**								
Hu 2020	44.45	4.39	50	40.48	4.01	50	15.5%	3.97 [2.32, 5.62]
Li 2020	45.62	3.12	40	41.58	1.31	40	16.9%	4.04 [2.99, 5.09]
Shi 2019	45.62	3.12	20	41.56	1.64	20	15.8%	4.06 [2.52, 5.60]
Wu 2021	44.98	2.3	44	42.03	2.41	44	17.0%	2.95 [1.97, 3.93]
Xiong 2018	39.79	0.63	25	33.46	0.29	25	17.9%	6.33 [6.06, 6.60]
Yang 2017	44.31	2.74	41	42.18	1.78	41	17.0%	2.13 [1.13, 3.13]
**Subtotal (95% CI)**			**220**			**220**	**100.0%**	**3.93 [2.16, 5.70]**
Heterogeneity: Tau² = 4.53; Chi² = 117.63, df = 5 (P < 0.00001); I² = 96%								
Test for overall effect: Z = 4.36 (P < 0.0001)								
**1.2.2 Sensitivity analysis**								
Hu 2020	44.45	4.39	50	40.48	4.01	50	13.5%	3.97 [2.32, 5.62]
Li 2020	45.62	3.12	40	41.58	1.31	40	33.3%	4.04 [2.99, 5.09]
Shi 2019	45.62	3.12	20	41.56	1.64	20	15.4%	4.06 [2.52, 5.60]
Wu 2021	44.98	2.3	44	42.03	2.41	44	37.8%	2.95 [1.97, 3.93]
**Subtotal (95% CI)**			**154**			**154**	**100.0%**	**3.62 [3.02, 4.23]**
Heterogeneity: Tau² = 0.00; Chi² = 2.88, df = 3 (P = 0.41); I² = 0%								
Test for overall effect: Z = 11.72 (P < 0.00001)								

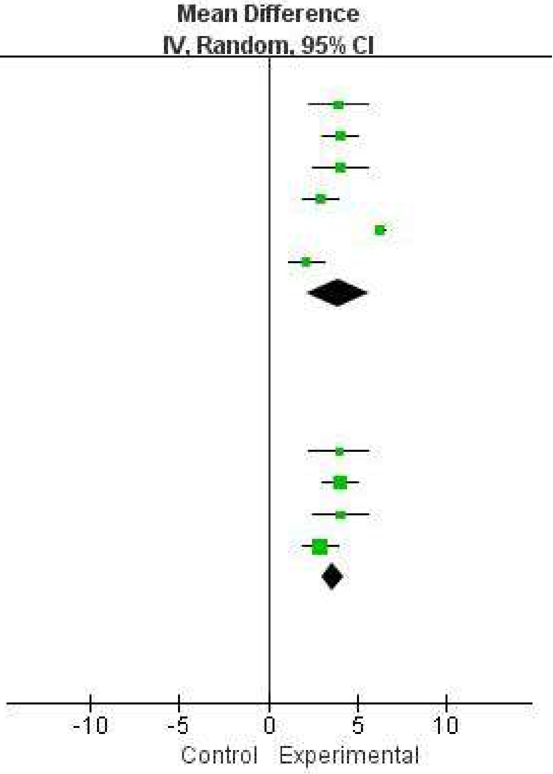

Supplementary Figure S3. Meta-analysis of CPMNCV

Study or Subgroup	Experimental Mean	Experimental SD	Experimental Total	Control Mean	Control SD	Control Total	Weight	Mean Difference IV, Random, 95% CI
**1.3.1 Common peroneal sensory nerve conduction velocity**								
Hu 2020	33.81	3.31	50	31.09	3.04	50	12.4%	2.72 [1.47, 3.97]
Li 2020	34.48	2.13	40	30.25	0.97	40	18.6%	4.23 [3.50, 4.96]
Shi 2019	34.48	2.13	20	30.48	1.23	20	14.2%	4.00 [2.92, 5.08]
Wu 2021	34.02	3.01	44	31.04	3.16	44	12.0%	2.98 [1.69, 4.27]
Xiong 2018	38.48	0.23	25	34.78	0.44	25	24.3%	3.70 [3.51, 3.89]
Yang 2017	33.42	1.55	41	31.22	1.85	41	18.4%	2.20 [1.46, 2.94]
**Subtotal (95% CI)**			**220**			**220**	**100.0%**	**3.36 [2.74, 3.98]**
Heterogeneity: Tau² = 0.40; Chi² = 20.99, df = 5 (P = 0.0008); I² = 76%								
Test for overall effect: Z = 10.61 (P < 0.00001)								
**1.3.2 Sensitivity analysis**								
Hu 2020	33.81	3.31	50	31.09	3.04	50	8.6%	2.72 [1.47, 3.97]
Li 2020	34.48	2.13	40	30.25	0.97	40	19.9%	4.23 [3.50, 4.96]
Shi 2019	34.48	2.13	20	30.48	1.23	20	11.0%	4.00 [2.92, 5.08]
Wu 2021	34.02	3.01	44	31.04	3.16	44	8.2%	2.98 [1.69, 4.27]
Xiong 2018	38.48	0.23	25	34.78	0.44	25	52.3%	3.70 [3.51, 3.89]
**Subtotal (95% CI)**			**179**			**179**	**100.0%**	**3.70 [3.30, 4.09]**
Heterogeneity: Tau² = 0.07; Chi² = 5.92, df = 4 (P = 0.21); I² = 32%								
Test for overall effect: Z = 18.28 (P < 0.00001)								

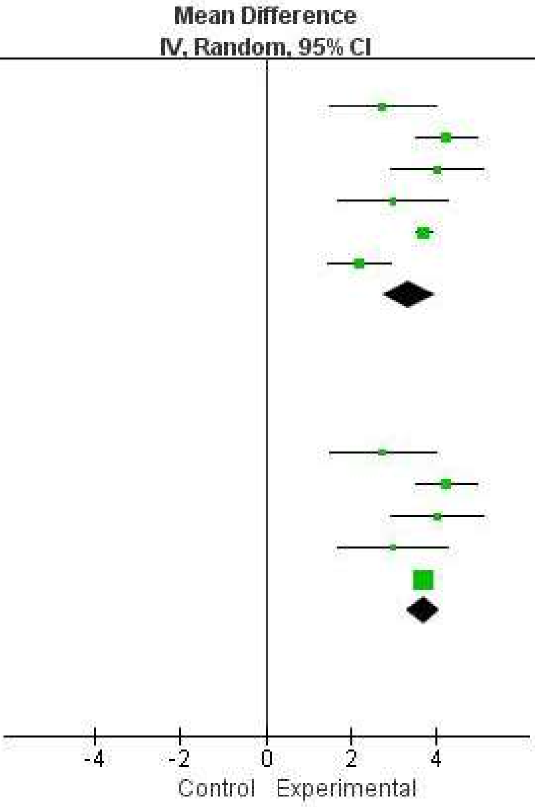

Supplementary Figure S4. Meta-analysis of CPSNCV

Study or Subgroup	Experimental Mean	Experimental SD	Experimental Total	Control Mean	Control SD	Control Total	Weight	Mean Difference IV, Random, 95% CI
**1.4.1 Median motor nerve conduction velocity**								
Hu 2020	57.92	5.77	50	50.68	5.04	50	9.4%	7.24 [5.12, 9.36]
Li 2020	58.41	2.99	40	52.64	2.21	40	17.3%	5.77 [4.62, 6.92]
Shi 2019	58.62	2.95	20	53.46	2.23	20	12.9%	5.16 [3.54, 6.78]
Wu 2021	58.03	3.09	44	54.2	2.96	44	16.1%	3.83 [2.57, 5.09]
Xiong 2018	60.74	0.17	25	56.48	0.91	25	25.1%	4.26 [3.90, 4.62]
Yang 2017	57.81	2.05	41	54.28	2.46	41	19.1%	3.53 [2.55, 4.51]
**Subtotal (95% CI)**			**220**			**220**	**100.0%**	**4.71 [3.90, 5.52]**
Heterogeneity: Tau² = 0.65; Chi² = 17.47, df = 5 (P = 0.004); I² = 71%								
Test for overall effect: Z = 11.34 (P < 0.00001)								
**1.4.2 Sensitivity analysis**								
Hu 2020	57.92	5.77	50	50.68	5.04	50	18.5%	7.24 [5.12, 9.36]
Li 2020	58.41	2.99	40	52.64	2.21	40	51.6%	5.77 [4.62, 6.92]
Shi 2019	58.62	2.95	20	53.46	2.23	20	29.9%	5.16 [3.54, 6.78]
**Subtotal (95% CI)**			**110**			**110**	**100.0%**	**5.86 [4.90, 6.82]**
Heterogeneity: Tau² = 0.12; Chi² = 2.36, df = 2 (P = 0.31); I² = 15%								
Test for overall effect: Z = 11.98 (P < 0.00001)								

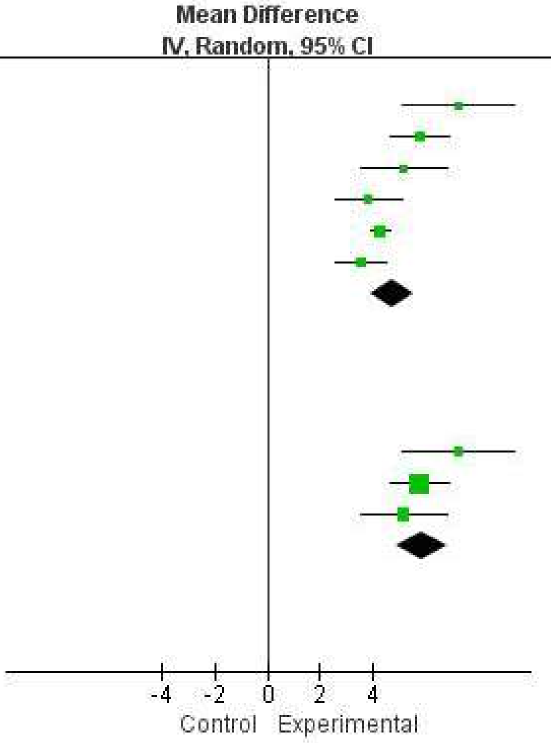

Supplementary Figure S5. Meta-analysis of MMNCV

	Experimental			Control				Mean Difference
Study or Subgroup	Mean	SD	Total	Mean	SD	Total	Weight	IV, Random, 95% CI
**1.5.1 Median sensory nerve conduction velocity**								
Hu 2020	44.62	4.43	50	39.79	3.94	50	11.6%	4.83 [3.19, 6.47]
Li 2020	44.23	1.54	40	40.48	1.11	40	18.5%	3.75 [3.16, 4.34]
Shi 2019	45.32	1.55	20	41.42	1.31	20	16.6%	3.90 [3.01, 4.79]
Wu 2021	45.08	2.42	44	42.44	2.51	44	15.6%	2.64 [1.61, 3.67]
Xiong 2018	57.57	0.54	25	55.48	0.45	25	19.8%	2.09 [1.81, 2.37]
Yang 2017	44.51	1.49	41	42.71	1.63	41	18.0%	1.80 [1.12, 2.48]
**Subtotal (95% CI)**			**220**			**220**	**100.0%**	**3.05 [2.19, 3.90]**
Heterogeneity: Tau² = 0.94; Chi² = 47.22, df = 5 (P < 0.00001); I² = 89%								
Test for overall effect: Z = 7.00 (P < 0.00001)								
**1.5.2 Sensitivity analysis**								
Hu 2020	44.62	4.43	50	39.79	3.94	50	12.7%	4.83 [3.19, 6.47]
Li 2020	44.23	1.54	40	40.48	1.11	40	36.9%	3.75 [3.16, 4.34]
Shi 2019	45.32	1.55	20	41.42	1.31	20	27.1%	3.90 [3.01, 4.79]
Wu 2021	45.08	2.42	44	42.44	2.51	44	23.4%	2.64 [1.61, 3.67]
**Subtotal (95% CI)**			**154**			**154**	**100.0%**	**3.67 [2.99, 4.34]**
Heterogeneity: Tau² = 0.23; Chi² = 6.08, df = 3 (P = 0.11); I² = 51%								
Test for overall effect: Z = 10.65 (P < 0.00001)								

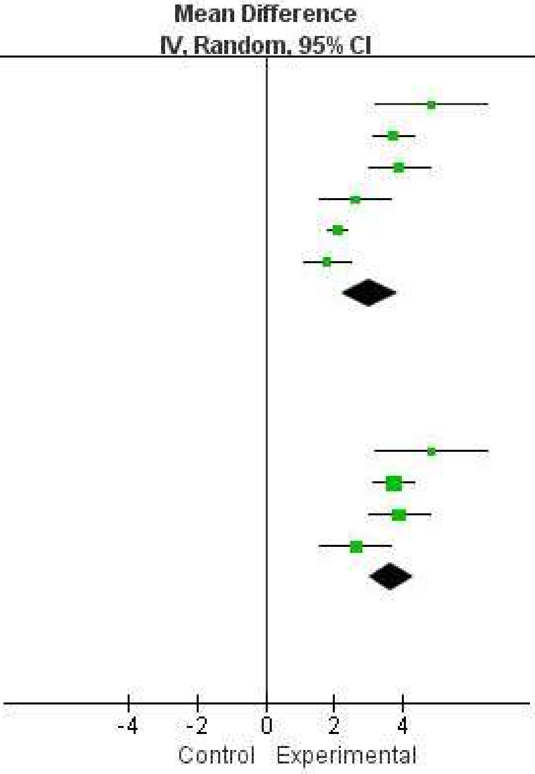

Supplementary Figure S6. Meta-analysis of MSNCV

Study or Subgroup	Experimental Mean	Experimental SD	Experimental Total	Control Mean	Control SD	Control Total	Weight	Mean Difference IV, Random, 95% CI
**1.6.1 Fasting blood glucose**								
Hu 2020	4.81	0.47	50	6.28	0.61	50	24.5%	-1.47 [-1.68, -1.26]
Sun 2023	5.56	0.67	45	6.68	0.59	43	21.6%	-1.12 [-1.38, -0.86]
Wu 2021	5.53	0.55	44	6.69	0.62	44	22.6%	-1.16 [-1.40, -0.92]
Yang 2017	4.84	0.22	41	5.87	0.14	41	31.3%	-1.03 [-1.11, -0.95]
**Subtotal (95% CI)**			**180**			**178**	**100.0%**	**-1.19 [-1.40, -0.98]**
Heterogeneity: Tau² = 0.03; Chi² = 14.70, df = 3 (P = 0.002); I² = 80%								
Test for overall effect: Z = 11.13 (P < 0.00001)								
**1.6.2 Sensitivity analysis**								
Hu 2020	4.81	0.47	50	6.28	0.61	50	36.3%	-1.47 [-1.68, -1.26]
Sun 2023	5.56	0.67	45	6.68	0.59	43	31.1%	-1.12 [-1.38, -0.86]
Wu 2021	5.53	0.55	44	6.69	0.62	41	32.5%	-1.16 [-1.41, -0.91]
**Subtotal (95% CI)**			**139**			**134**	**100.0%**	**-1.26 [-1.49, -1.03]**
Heterogeneity: Tau² = 0.03; Chi² = 5.35, df = 2 (P = 0.07); I² = 63%								
Test for overall effect: Z = 10.83 (P < 0.00001)								

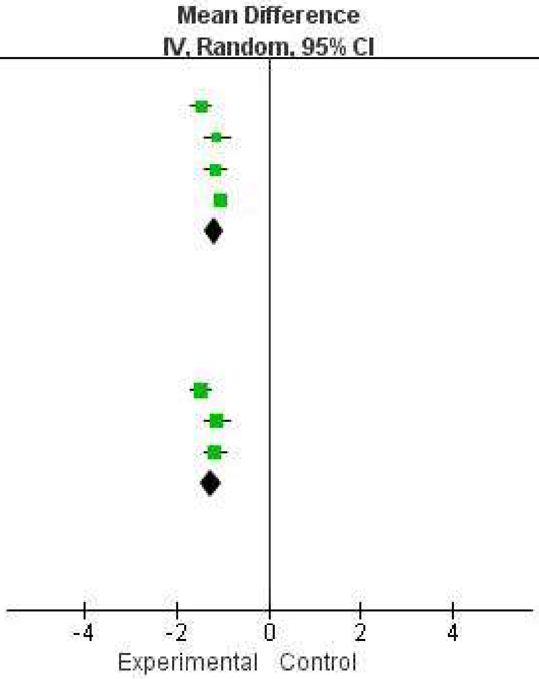

Supplementary Figure S7. Meta-analysis of FPG

	Experimental			Control				Mean Difference
Study or Subgroup	Mean	SD	Total	Mean	SD	Total	Weight	IV, Random, 95% CI
Hu 2020	10.49	1.02	50	11.82	1.21	50	3.9%	-1.33 [-1.77, -0.89]
Sun 2023	8.34	0.62	45	9.64	0.71	43	9.7%	-1.30 [-1.58, -1.02]
Wu 2021	10.48	0.78	44	11.9	0.86	44	6.4%	-1.42 [-1.76, -1.08]
Yang 2017	10.52	0.28	41	11.88	0.15	41	79.9%	-1.36 [-1.46, -1.26]
**Total (95% CI)**			**180**			**178**	**100.0%**	**-1.36 [-1.44, -1.27]**

Heterogeneity: Tau² = 0.00; Chi² = 0.31, df = 3 (P = 0.96); I² = 0%

Test for overall effect: Z = 30.59 (P < 0.00001)

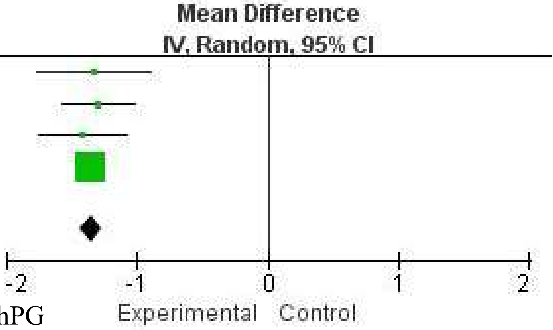

Supplementary Figure S8. Meta-analysis of 2hPG

Study or Subgroup	Experimental Mean	Experimental SD	Experimental Total	Control Mean	Control SD	Control Total	Weight	Mean Difference IV, Random, 95% CI
Hu 2020	5.84	0.52	50	6.72	0.63	50	24.6%	-0.88 [-1.11, -0.65]
Sun 2023	5.94	0.59	45	7.01	0.55	43	23.4%	-1.07 [-1.31, -0.83]
Wu 2021	6.12	0.89	44	7.03	0.95	44	13.0%	-0.91 [-1.29, -0.53]
Yang 2017	5.87	0.31	41	6.61	0.15	41	39.0%	-0.74 [-0.85, -0.63]
**Total (95% CI)**			**180**			**178**	**100.0%**	**-0.87 [-1.04, -0.71]**

Heterogeneity: Tau² = 0.01; Chi² = 6.89, df = 3 (P = 0.08); I² = 56%

Test for overall effect: Z = 10.48 (P < 0.00001)

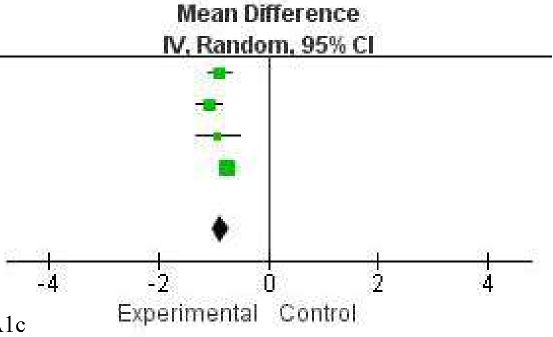

Supplementary Figure S9. Meta-analysis of HbA1c

Supplementary Table S1. Search strategies

**PubMed**	**#1:** “diabetes mellitus”[MeSH] OR “diabetes mellitus” [Title/Abstract]<br><br>**#2:** “peripheral nervous system diseases” [MeSH] OR “peripheral nervous system disease” [Title/Abstract] OR “peripheral neuropathy” [Title/Abstract] OR “peripheral nerve disease” [Title/Abstract] OR “peripheral nervous system disorder” [Title/Abstract]<br><br>**#3:** “diabetic neuropathies” [MeSH] OR “diabetic neuropathy” [Title/Abstract] OR “diabetic autonomic neuropathy” [Title/Abstract] OR “diabetic neuralgia” [Title/Abstract] OR “diabetic mononeuropathy” [Title/Abstract] OR “diabetic amyotrophy” [Title/Abstract] OR “diabetic polyneuropathy” [Title/Abstract]<br><br>**#4:** "dapagliflozin"[MeSH] OR "dapagliflozin"[Title/Abstract] OR "Forxiga"[MeSH] OR "Farxiga"[MeSH] OR "(2S,3R,4R,5S,6R)-2-(4-chloro-3-(4-ethoxybenzyl)phenyl)-6- (hydroxymethyl)tetrahydro-2H-pyran-3,4,5-triol"[MeSH] OR "2-(3-(4-ethoxybenzyl) -4-chlorophenyl)-6-hydroxymethyltetrahydro-2H-pyran-3,4,5-triol"[MeSH] OR "BMS 512148"[MeSH] OR "BMS512148"[MeSH] OR "BMS-512148"[MeSH]<br><br>**#5:**"mecobalamin"[MeSH] OR "mecobalamin"[Title/Abstract] OR “methylvitamin B12”[MeSH] OR “CH3-B12”[MeSH] OR “methylcobalamin” [MeSH] OR “methylcobalamine” [MeSH] OR “mecobalamin, Co-methyl-14C-labeled” [MeSH] OR “mecobalamin, Co-methyl-13C-labeled” [MeSH] OR “mecobalamin, (13beta)-isomer” [MeSH] OR “mecobalamin, 3H-labeled” [MeSH] OR “mecobalamin monohydrate” [MeSH] OR “mecobalamin, 57Co-labeled”[MeSH]
	((#1 AND #2) OR #3) AND (#4 AND #5)
**Embase**	**#1:** ‘diabetes mellitus’/exp OR ‘diabetes mellitus’:ab,ti<br><br>**#2:** 'peripheral nervous system diseases'/exp OR ‘peripheral nervous system disease’: ab,ti OR ‘peripheral neuropathy’:ab,ti OR ‘peripheral nerve disease’:ab,ti OR 'peripheral nervous system disorder':ab,ti<br><br>**#3:**‘diabetic neuropathies’/exp OR ‘diabetic neuropathy’:ab,ti OR ‘diabetic autonomic neuropathy’:ab,ti OR ‘diabetic neuralgia’:ab,ti OR ‘diabetic mononeuropathy’:ab,ti OR ‘diabetic amyotrophy’:ab,ti OR ‘diabetic polyneuropathy’:ab,ti<br><br>**#4:** ‘dapagliflozin’/exp OR ‘dapagliflozin’:ab,ti OR ‘Forxiga’/exp OR ‘Farxiga’/exp OR‘(2S,3R,4R,5S,6R)-2-(4-chloro-3-(4-ethoxybenzyl) phenyl)-6- (hydroxymethyl)tetrahydro-2H-pyran-3,4,5-triol’/exp OR ‘2-(3-(4-ethoxybenzyl)-4-chlorophenyl)-6-hydroxymethyltetrahydro-2H-pyran-3,4,5-triol’/exp OR ‘BMS 512148’/exp OR ‘BMS512148’/exp OR ‘BMS-512148’/exp

	**#5:**'mecobalamin'/exp OR 'mecobalamin':ab,ti OR 'methylvitamin B12'/exp OR 'CH3-B12'/exp OR 'methylcobalamin'/exp OR 'methylcobalamine'/exp OR 'mecobalamin, Co-methyl-14C-labeled'/exp OR 'mecobalamin, Co-methyl-13C-labeled'/exp OR 'mecobalamin, (13beta)-isomer'/exp OR 'mecobalamin, 3H-labeled'/exp OR 'mecobalamin monohydrate'/exp OR 'mecobalamin, 57Co-labeled'/exp
	((#1 AND #2) OR #3) AND (#4 AND #5)
**Cochrane Library**	**#1:** MeSH descriptor: [diabetes mellitus] explode all trees<br>**#2:** MeSH descriptor: [peripheral nervous system diseases] explode all trees<br>**#3:** MeSH descriptor: [diabetic neuropathies] explode all trees<br>**#4:** "peripheral nervous system disease" OR "peripheral neuropathy" OR "peripheral nerve disease" OR "peripheral nervous system disorder": ti,ab,kw<br>**#5:** "diabetic neuropathy" OR "diabetic autonomic neuropathy" OR "diabetic neuralgia" OR "diabetic mononeuropathy" OR "diabetic amyotrophy" OR "diabetic polyneuropathy": ti,ab,kw<br>**#6:** ((#1 AND #2) OR #3) OR #4 OR #5<br>**#7:** MeSH descriptor: [dapagliflozin] explode all trees<br>**#8:** MeSH descriptor: [Forxiga] explode all trees<br>**#9:** MeSH descriptor: [Farxiga] explode all trees<br>**#10:** MeSH descriptor: [(2S,3R,4R,5S,6R)-2-(4-chloro-3-(4-ethoxybenzyl)phenyl)-6- (hydroxymethyl)tetrahydro-2H-pyran-3,4,5-triol] explode all trees<br>**#11:** MeSH descriptor: [2-(3-(4-ethoxybenzyl)-4-chlorophenyl)-6-hydroxymethyltetrahydro-2H-pyran-3,4,5-triol] explode all trees<br>**#12:** MeSH descriptor: [BMS 512148] explode all trees<br>**#13:** MeSH descriptor: [BMS-512148] explode all trees<br>**#14:** #7 OR #8 OR #9 OR #10 OR #11 OR #12 OR #13<br>**#15:** MeSH descriptor: [mecobalamin] explode all trees<br>**#16:** MeSH descriptor: [methylvitamin B12] explode all trees<br>**#17:** MeSH descriptor: [CH3-B12] explode all trees<br>**#18:** MeSH descriptor: [methylcobalamin] explode all trees<br>**#19:** MeSH descriptor: [methylcobalamine] explode all trees<br>**#20:** MeSH descriptor: [mecobalamin, Co-methyl-14C-labeled] explode all trees<br>**#21:** MeSH descriptor: [mecobalamin, Co-methyl-13C-labeled] explode all trees<br>**#22:** MeSH descriptor: [mecobalamin, (13beta)-isomer] explode all trees<br>**#23:** MeSH descriptor: [mecobalamin, 3H-labeled] explode all trees<br>**#24:** MeSH descriptor: [mecobalamin monohydrate] explode all trees<br>**#25:** MeSH descriptor: [mecobalamin, 57Co-labeled] explode all trees<br>**#26:** #15 OR #16 OR #17 OR #18 OR #19 OR #20 OR #21OR #22 OR #23OR #24 OR #25
	**(**#6 AND #14 AND #26**)**
**CNKI**	**#1:** TKA%='糖尿病' AND TKA%= '周围神经病变'<br>**#2:** TKA%='甲钴胺' OR TKA%='弥可保' OR TKA%='硫辛酸'

	**#3:** TKA%='达格列净' OR TKA%='安达唐' OR TKA%='福适佳'<br>**#4:** #1 AND #2 AND #3

Supplementary Table S2. Data analysis of included trials

Outcome or Subgroup	Studies	Participants	Statistical Method	Effect Estimate
1.1 OER	5	438	Odds Ratio (M-H, Fixed, 95% CI)	5.05[2.60, 9.81]
1.2 CPMNCV	6	440	Mean Difference (IV, Random, 95% CI)	3.93 [2.16, 5.70]
1.3 CPSNCV	6	440	Mean Difference (IV, Random, 95% CI)	3.36 [2.74, 3.98]
1.4 MMNCV	6	440	Mean Difference (IV, Random, 95% CI)	4.71[3.90, 5.52]
1.5 MSNCV	6	440	Mean Difference (IV, Random, 95% CI)	3.05 [2.19, 3.90]
1.6 FPG	3	276	Mean Difference (IV, Random, 95% CI)	-1.26 [-1.49, -1.03]
1.7 2hPG	4	358	Mean Difference (IV, Random, 95% CI)	-1.36 [-1.44, -1.27]
1.8 HbA1c	4	358	Mean Difference (IV, Random, 95% CI)	-0.87 [-1.04, -0.71]
1.9 RAE	1	88	Odds Ratio (M-H, Fixed, 95% CI)	0.37 [0.07, 2.03]

Note: IV, inverse variance; CI, confidence interval; M-H, mantel-haenszel;OER, overall effective rate; CPMNCV, common peroneal motor nerve conduction velocity; CPSNCV, common peroneal sensory nerve conduction velocity; MMNCV, median motor nerve conduction velocity; MSNCV, median sensory nerve conduction velocity; FPG, fasting blood glucose; 2hPG, 2-h postprandial blood glucose; HbA1c, glycosylated hemoglobin; RAE, rate of adverse events.

Supplementary Table S3. GRADE evidence profile

Outcome	Number of Studies	Study Design	Risk of Bias	Inconsistency	Indirectness	Imprecision	Publication Bias	Pooled Effect	I^2	Certainty of the evidence (GRADE)
OER	5 (438)	RCT	Serious	Not serious	Not serious	Not serious	Undetected	OR:5.05[2.60, 9.81]	0%	Moderate
CPMNCV	6 (440)	RCT	Serious	Serious*	Not serious	Not serious	Undetected	MD:3.93 [2.16, 5.70]	96%	Low
CPSNCV	6 (440)	RCT	Serious	Serious*	Not serious	Not serious	Undetected	MD:3.36 [2.74, 3.98]	76%	Low
MMNCV	6 (440)	RCT	Serious	Serious*	Not serious	Not serious	Undetected	MD:4.71[3.90, 5.52]	71%	Low
MSNCV	6 (440)	RCT	Serious	Serious*	Not serious	Not serious	Undetected	MD:3.05 [2.19, 3.90]	89%	Low
FPG	3 (276)	RCT	Serious	Serious*	Not serious	Not serious	Undetected	MD:-1.26 [-1.49, -1.03]	80%	Low
2hPG	4 (358)	RCT	Serious	Not serious	Not serious	Not serious	Undetected	MD:-1.36 [-1.44, -1.27]	0%	Moderate
HbA1c	4 (358)	RCT	Serious	Serious*	Not serious	Not serious	Undetected	MD:-0.87 [-1.04, -0.71]	56%	Low
RAE	1 (88)	RCT	Serious	Undetected	Not serious	Serious†	Undetected	OR:0.37 [0.07, 2.03]	NA	Low

Note: GRADE: Working Group grades of evidence.
High quality: Further research is very unlikely to change our confidence in the estimate of effect.
Moderate quality: Further research is likely to have an important impact on our confidence in the estimate of effect and may change the estimate.
Low quality: Further research is very likely to have an important impact on our confidence in the estimate of effect and is likely to change the estimate.
Very low quality: We are very uncertain about the estimate.
*Downgraded as the intrastudy inconsistency was significant (I^2>50%).
†Downgraded for continuous variables if the sample size was <100.
GRADE indicates Grading of Recommendations, Assessment, Development and Evaluation; RCT, randomized controlled trial; OR,Odds Ratio; MD, mean difference; NA, not applicable; OER, overall effective rate; CPMNCV, common peroneal motor nerve conduction velocity; CPSNCV, common peroneal sensory nerve conduction velocity; MMNCV, median motor nerve conduction velocity; MSNCV, median sensory nerve conduction velocity; FPG, fasting blood glucose; 2hPG, 2-h postprandial blood glucose; HbA1c, glycosylated hemoglobin; RAE, rate of adverse events.
